# Ecotones in the Spotlight—Habitat Selection of the Golden Jackal (*Canis aureus* Linnaeus, 1758) in the Agricultural Landscapes of Central Europe

**DOI:** 10.3390/ani15050760

**Published:** 2025-03-06

**Authors:** Dorottya Karolin Gaál, Miklós Heltai, Gyula Sándor, Gergely Schally, Erika Csányi

**Affiliations:** 1 Fauna and Flora Foundation, 7561 Nagybajom, Hungary; gaal.dorottya.karolin@stud.uni-mate.hu; 2 Department of Wildlife Biology and Management, Institute for Wildlife Management and Nature Conservation, Hungarian University of Agriculture and Life Sciences, 2100 Gödöllő, Hungary; heltai.miklos.gabor@uni-mate.hu (M.H.); schally.gergely.tibor@uni-mate.hu (G.S.); 3 Institute of Wildlife Biology and Management, University of Sopron, 9400 Sopron, Hungary; sandor.gyula@uni-sopron.hu

**Keywords:** *Canis aureus*, home range, habitat use, ecotones, agro-ecosystem

## Abstract

The large-scale expansion of the golden jackal (*Canis aureus*) across Europe in recent decades has been strongly influenced by its successful space and habitat use. In this study, we analyzed the habitat selection of seven golden jackals tracked with GPS collars between 15 March 2021 and 25 November 2022 in a predominantly agricultural landscape in the southwestern part of the Pannonian Basin, Central Europe. Animals were tracked for an average of 29 weeks, and GPS collars recorded a total of 29,840 hourly localization points, which were compared to a high-resolution land cover dataset. We found that golden jackals maintain smaller home ranges in agricultural landscapes than in more pristine environments. Based on Jacobs’ index values calculated for monthly habitat preferences and the distribution of distances from land cover edges, we also found that preferences for the various habitat types differed significantly among individuals. Most of the time, golden jackals stayed near the edges of forests, agricultural lands, and shrublands, while they stayed away from artificial areas, wetlands, and water bodies. Forests and shrublands providing cover and safety were generally preferred by the golden jackals, especially during breeding and pup-rearing periods, while there was a strong avoidance of agricultural lands in general. Overall, our findings suggest that despite individual differences in the availability of habitat types within home ranges, forest–agricultural ecotones with relative proximity to food and shelter play a key role in the habitat selection of golden jackals.

## Simple Summary

In recent decades, the golden jackal (*Canis aureus*) has expanded considerably across Europe, raising numerous ecological, wildlife management, and legal concerns. In this study, we examined one of the main drivers of this successful expansion, the habitat use of the species. We found that the golden jackal has no clear preference towards one habitat type—complying with its generalist nature—however, it avoids open agricultural lands. Our results further prove that habitat edges in agro-ecosystems offering shelter and proximity to food resources represent the most sought-after habitats for the species. Understanding the importance of forest–agricultural ecotones in the habitat use of the golden jackal is crucial for developing an informed conservation and wildlife management opinion on the species.

## 1. Introduction

The large-scale ongoing range expansion of the golden jackal (*Canis aureus* Linnaeus, 1758) across the European continent raises numerous conservation [1,2,3], management [4], and legal concerns [4,5]. This phenomenon is considered the largest population explosion documented on the continent [6], with the successful space use of this medium-sized carnivore playing a crucial role, referring to its capacity to exploit resources in fragmented and diverse habitats while minimizing risks from varying anthropogenic pressures [7,8,9,10].

Several factors may contribute to the spread of a species, and climate change [11] and land-use changes [12] are among the main drivers. In the case of the golden jackal, researchers attribute its rapid expansion primarily to human-induced habitat and land-use changes, increased food availability, and to the decline and fragmentation of the gray wolf (*Canis lupus*) population in the 20th century [6,7,13]. The gray wolf population, however, has shown an increasing trend across Europe in recent decades [14,15]; nevertheless, golden jackals have continued to expand, particularly in areas where wolves are still absent [5,13]. Additionally, studies further suggest that its high dietary flexibility and social behavior adaptability also play a significant role in the species’ successful expansion [16,17].

The golden jackal is highly adaptable, enabling it to occupy a wide range of habitats [8,10,18,19]. While primarily a resident of lowland steppe regions, it is the most desert-adapted jackal, capable of surviving in water-scarce areas [20]. Typically, the distribution of the species is limited by natural factors, such as deep and extensive snow cover, extreme frost, and large continuous forest coverage [6]. However, recent evidence indicates that the golden jackal is even capable of surviving the harsh conditions of the far north in winter [21]. In Africa, the species ranges from sea level in Eritrea to elevations of 3500 m in the Bale Mountains [22]. In Europe, previous observations showed that the golden jackal avoided habitats above 500 m in elevation, preferring flat or gently sloping areas, particularly agricultural landscapes, shrublands, and open terrains [6]. Nevertheless, recent sightings increasingly report their presence in high mountain regions [23]. Although the golden jackal thrives in human-dominated landscapes with abundant food sources [8,19,24,25,26], studies also demonstrated that the species can successfully expand into regions with higher forest cover and lower anthropogenic food availability [27].

The golden jackal is an opportunistic species [7,8,28], occasionally scavenging carrion, prey of other carnivores, and human food waste but primarily predating on small mammals, particularly rodents [20,24,29,30,31]. Additionally, they secondarily feed on wild ungulates and plants [16,32].

The home range size in golden jackals varies among individuals depending on sex, age, and biological period [10] and may as well be affected by the proximity of villages [25]. The home range of golden jackals tracked across various habitats can cover areas from 1 km^2^ to nearly 70 km^2^, with core areas ranging between 1 km^2^ and 10 km^2^ [8,10,18,19,25,33,34].

The habitat selection of individuals is influenced by various factors, such as habitat characteristics, disturbances caused by human activities, food availability, and social structure [6,7,8,17,35,36]. Golden jackals also adjust their habitat selection strategies according to agricultural intensity and the availability of suitable habitats. In areas with more intensive agricultural practices, their preference for shrubland and heterogeneous agricultural vegetation increases, highlighting their high intra-individual variability in space use [7,10]. Observations further show that the golden jackal favors intensively cultivated agricultural areas [6,24] but exhibits a circadian movement and habitat selection pattern to avoid encounters with humans [8,24,33,37]. During the daytime, golden jackals primarily stay in areas with dense vegetation and typically search for food at night [8,24].

While the importance of ecotonal environments in ungulate herbivores has long been highlighted [38], generalist species can also benefit from habitat fragmentation induced by agriculture, as they are well able to exploit habitat edges [39,40]. These human-generated, local-scale transitional areas, which may also be referred to as anthropogenic ecotones [41], shape mesocarnivores’ spatial behavior, as they serve as travel corridors within the landscape [42] and offer improved foraging conditions [43]. In forest–farmland ecotones, the activity of the red fox (*Vulpes vulpes*), for instance, is characterized by an intense search for its principal prey, small mammals [40], of which species richness and abundance is higher in ecotones than in open habitats [44]. Elevated wader nest predation rates have also been recently attributed to the species near forest edges in wet grasslands fragmented by forests [45]. The lesser spotted eagle (*Clanga pomarine*), a generalist avian predator species, has also been found to prefer more diverse territories and to forage near forest edges [46]. Golden jackals also tend to stay close to forest–agriculture ecotones [10], the exact drivers for which have not yet been identified.

Our study aimed to examine the habitat selection and space-use patterns of golden jackals in an agricultural environment. We hypothesized the following: Hypothesis 1 (H1): The golden jackal’s home range size will be smaller in agricultural habitats compared to pristine environments due to the increased availability of food resources provided by a denser population of small rodents in agricultural areas. Prediction 1 (P1): Golden jackals will exhibit smaller home ranges in agricultural landscapes compared to those documented in pristine habitats [25]. Hypothesis 2 (H2): The golden jackal prefers forests and shrublands for permanent cover and safety over agricultural lands with increased exposure to potential threats. Prediction 2 (P2): Golden jackals will select forest and shrubland habitats providing permanent cover over agricultural lands [8,24,33,37]. Prediction 3 (P3): Golden jackals will demonstrate a strong avoidance of agricultural lands in their habitat selection [8,24,33,37]. Hypothesis 3 (H3): Forest–agricultural ecotones play a key role in the habitat use of golden jackals, providing essential shelter and foraging opportunities. Prediction 4 (P4): Golden jackals will show a higher frequency of use in forest–agricultural ecotones compared to other habitat types [8,10].

## 2. Materials and Methods

### 2.1. Study Area

**Figure 1:**
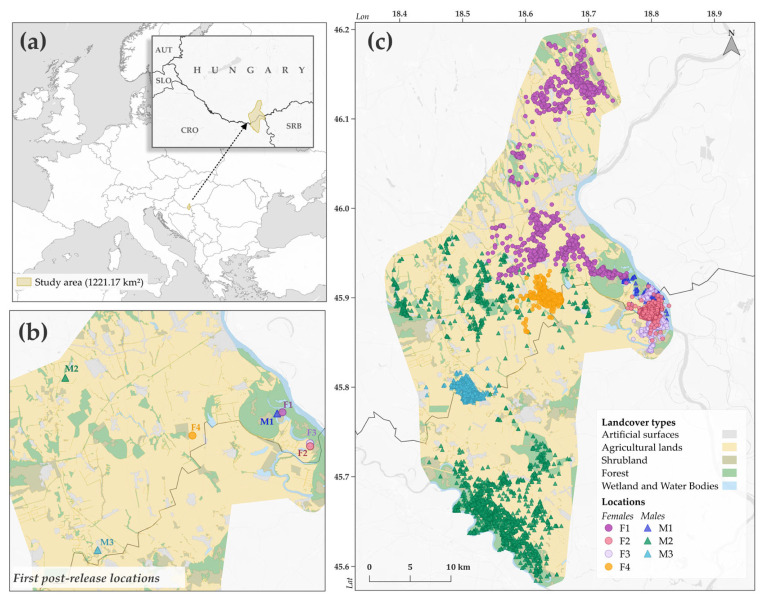
(**a**) Location of the study area within which 4 female and 3 male golden jackals (*Canis aureus*) were tracked with GPS collars. (**b**) First post-release localization points recorded by the collars. (**c**) The habitat structure of the study area indicating all localization points (*n* = 29,840) of seven GPS-tracked golden jackals.

The study was conducted in the southwestern part of the Pannonian Basin near the border of Hungary and Croatia. Based on the CORINE land cover (CLC) 2018 dataset [47] verified by more recent satellite imagery [48], the approximately 1220 km^2^ study area is characterized by a low forest and shrubland cover (14%, 7%), dominated by agricultural lands (70%) (Figure 1). The most important agricultural crops in the area are corn, winter wheat, autumn cereals, soybeans, and canola.

The composition of forests within the study area is highly variable. Along water bodies, vegetation is characterized by various willow (*Salix* spp.) and poplar (*Populus* spp.) species, as well as riverine woodlands interspersed with oaks (*Quercus* spp.) and ashes (*Fraxinus* spp.). Monospecific black locust (*Robinia pseudoacacia*) and European hornbeam (*Carpinus betulus*) forests are also present. Approximately 65% of the forests consist of hardwood species, while the remaining one-third comprises softwood riverine woodlands. A shrub layer is present in 70% of the forests, where the most prevalent shrub species include common dogwood (*Cornus sanguinea*), cornelian cherry (*Cornus mas*), various poplars, willows, ashes, and blackberry species (*Rubus* spp.) [49]. Shrublands found in the study area are wet and mesic pioneer scrub (most dominant species include common sallow (*Salix cinerea*), common hazel (*Corylus avellana*), European elderberry (*Sambucus nigra*)), dry and semi-dry pioneer scrub (dominated by hawthorn (*Crataegus monogyna*) and blackthorn (*Prunus spinosa*)) or riverine willow shrubs (*Salix* spp.) along water bodies [50].

Artificial surfaces (6%)—including CLC land cover classes of urban fabric, industrial, commercial and transport units; mine, dump and construction sites; artificial non-agricultural vegetated areas; and areas with complex cultivation patterns [47]—and wetlands and water bodies (3%) occupy relatively small areas. Whereas most of the area is intensively cultivated, Protected Natura 2000 sites cover 215 km^2^ mostly in the floodplains of the Danube and Drava rivers [51]. The golden jackal is the largest carnivore in the area (Figure 1).

### 2.2. Tagging and Data Collection

Seven golden jackals (4 females and 3 males) were captured in Hungary using baited box traps between 15 March 2021 and 25 November 2023 and fitted with Vertex Lite 1C Iridium GPS satellite collars (Vectronic Aerospace GmbH, Berlin, Germany; collar weight: 270 g). Based on dentition characteristics, body dimension, and fur coloration [52], three individuals were classified as juveniles (between 1 and 2 years; two female and one male) and four as adults (at least 2 years; two female and two male). Animals were tracked for 206 days on average (min = 63; max = 297) by the GPS collars recording hourly positions, resulting in 29,840 valid localization points in total (Figure 1, Table 1).

### 2.3. Data Analysis

The home range size of the golden jackals was calculated by different methods, namely the alpha concave hull (ACH) [53], the 95% minimum convex polygon (MCP95) [54], and the 95% kernel density estimation (KDE95) [55]. We also calculated the core areas of the individuals using the 50% kernel density estimator (KDE50) [55]. We used the adehabitatHR package [56] in R software (v4.4.1) for the MCP and KDE calculations, while the ACH areas were calculated in QGIS software (v3.38.3) [57].

For analyzing the habitat use of the seven tracked golden jackals, data on land cover types found within the study area were obtained from the CLC 2018 dataset [47]. Land cover types were verified based on more recent satellite imagery [48], then categories were grouped into five land cover types, namely artificial surfaces; agriculture; shrubland; forest; and wetland and water bodies. Habitat selection of the individuals was examined first by calculating the proportion of land cover categories within the individuals’ monthly home ranges (availability) delineated by the alpha concave hull (ACH) method. The ACH method for home range estimation was chosen, as we intended to include all valid data points in the habitat selection analysis but avoid overestimating the available habitats. We examined habitat preference by calculating an index of preference for each land cover category using Jacobs’ formula [58]:

D = (r_i_ − p_i_)/(r_i_ + p_i_ − 2 × r_i_ × p_i_)

where r_i_ is the proportion of localization points recorded in the specific habitat type and p_i_ is the proportion of that habitat type in the individuals’ home range. The resulting index (D) ranges between −1 (maximum avoidance) and 1 (maximum preference). For data visualization and statistical analysis, we used R software (v4.4.1) with the ggplot2 package [59].

To further explore the habitat use of the golden jackal, we evaluated how close they stayed to different landscape features (homogenous patches). For this purpose, we calculated distances to the nearest land cover features for all localization points in the study area expanded with a 10 km buffer zone. The expansion was important to ensure that valid distances could be calculated to the nearest land cover features for the outermost localization points, even if those features were located outside the original study area. In order to determine the availability of the distances that can actually be covered between landscape features, we established 250 m wide distance zones between the patches belonging to each habitat category by using multi-ring buffers in GIS, and we also calculated their areas. To explore if any circadian patterns characterize the golden jackals’ use of edge habitats, we also examined the hourly distances from the different landscape features. For these calculations, as well as for spatial data visualization, we used QGIS software, and data visualization was performed using R software with the *ggplot2* package and JASP software (v.0.19.3) [60].

## 3. Results

### 3.1. Home Range Size

The mean (±SD) home range size of the seven golden jackals was 131.8 ± 222.0 km^2^ (KDE95), varying significantly among the individuals (range: 2.5–526.8 km^2^) (Table 2). Home ranges estimated using MCP95 (145.1 ± 245.6 km^2^) and ACH (135.2 ± 231.2 km^2^) methods resulted in higher averages, as well as higher inter-individual variations (Table 2). Core areas calculated by the KDE50 method were much smaller (20.7 ± 34.6 km^2^), ranging between 0.3 and 76.6 km^2^ (Table 2).

**Figure 2:**
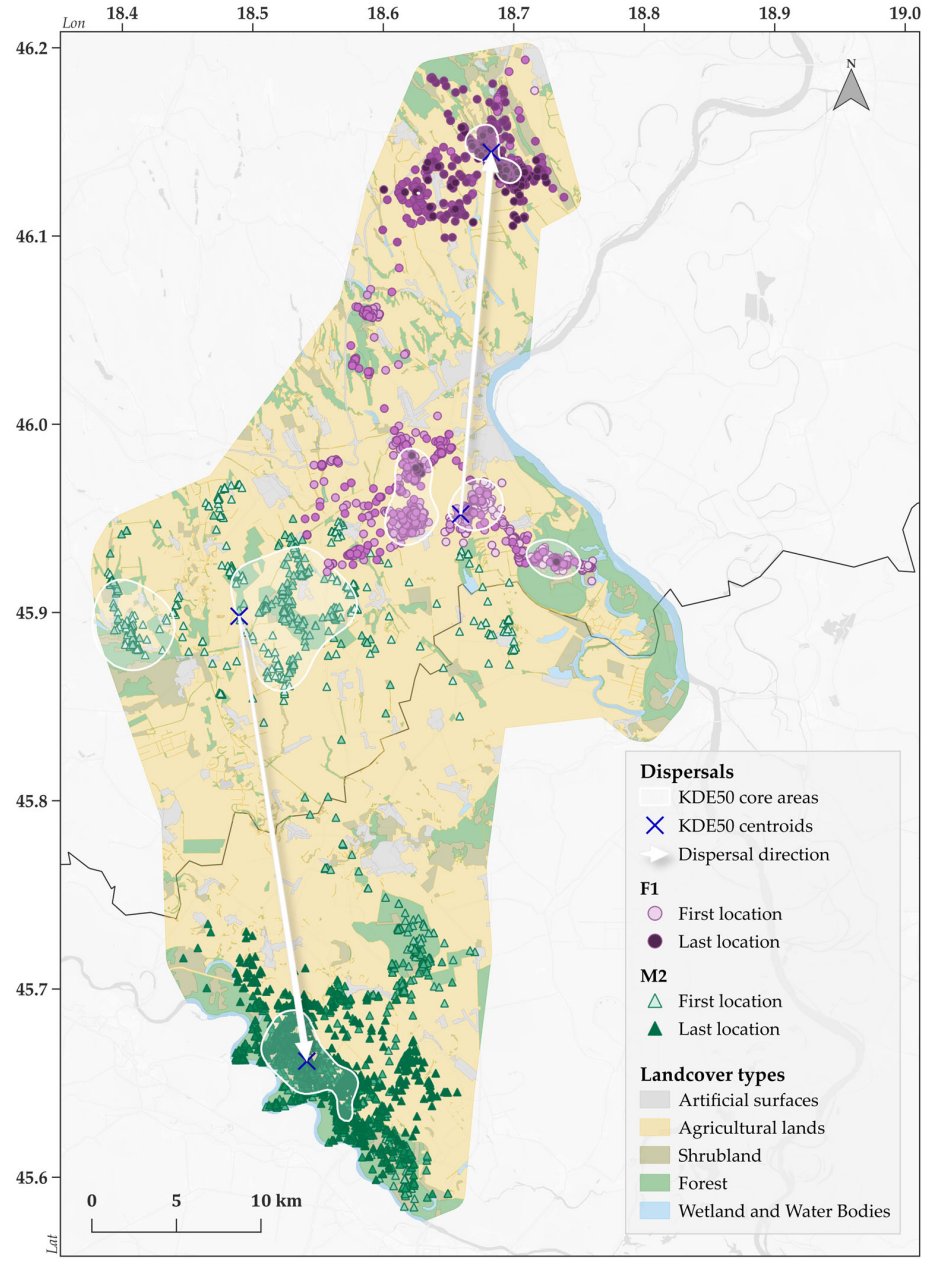
The dispersal of a female (F1) and male (M2) juvenile golden jackals in November and December of 2021.

F1 and M2 individuals showed particularly high home range and core area sizes concerning all calculation methods. Examining the movement patterns of the two juvenile individuals in more detail, it is safe to assume that they shifted their home ranges to new territories in November and December in 2021. The F1 individual established its new smaller home range (Table 3) further north, with 21.44 km between the centroids of its old and new core areas (KDE50). The M2 individual moved further south compared to its previous range, crossing the national border into the vicinity of the Drava River, eventually occupying a smaller home range and core area (Table 3), 26.58 km away from its previous territories (Figure 2).

The mean (±SD) home range size of resident golden jackals—which maintained a single home range throughout the study period—was substantially smaller (ACH: 17.6 ± 3.2 km^2^, MCP95: 9.3 ± 7.0 km^2^; KDE95: 4.4 ± 1.6 km^2^), and their core areas were all less than 1 km^2^ (KDE50: 0.5 ± 0.2 km^2^).

### 3.2. Habitat Selection

**Figure 3:**
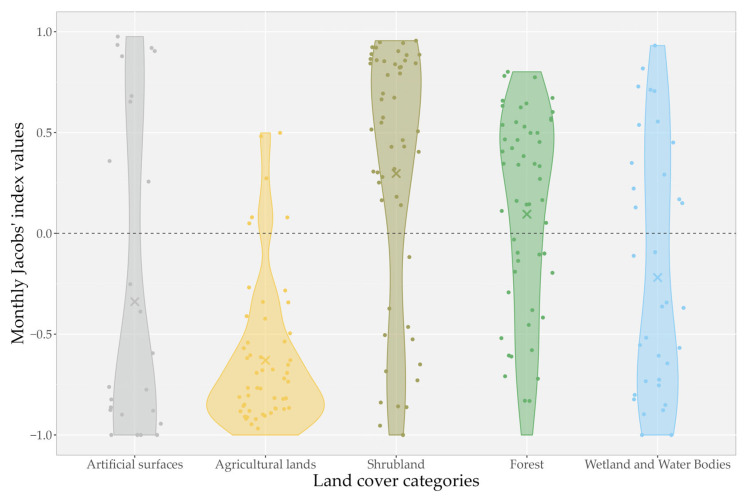
Habitat preferences of seven GPS-tracked golden jackals (*Canis aureus*) in the agricultural landscapes of the southwestern part of the Pannonian Basin based on monthly Jacobs’ index values of habitat selection. The violin plots depict the distribution of Jacobs’ index values for each land cover category; jittered points represent individual data values, while X symbols indicate the mean Jacobs’ index for each category.

In general, Jacobs’ index values of habitat selection calculated based on the monthly localization points of the individuals showed a strong avoidance of agricultural lands (*D_agr_* = −0.63 ± 0.37) and moderate avoidance of artificial surfaces (*D_art_* = −0.34 ± 0.78) and wetlands and water bodies (*D_ww_* = −0.22 ± 0.63). Shrubland and forest habitat types were generally preferred by the golden jackals on average (*D_shr_* = 0.30 ± 0.65; *D_for_* = 0.10 ± 0.52) (Figure 3).

**Figure 4:**
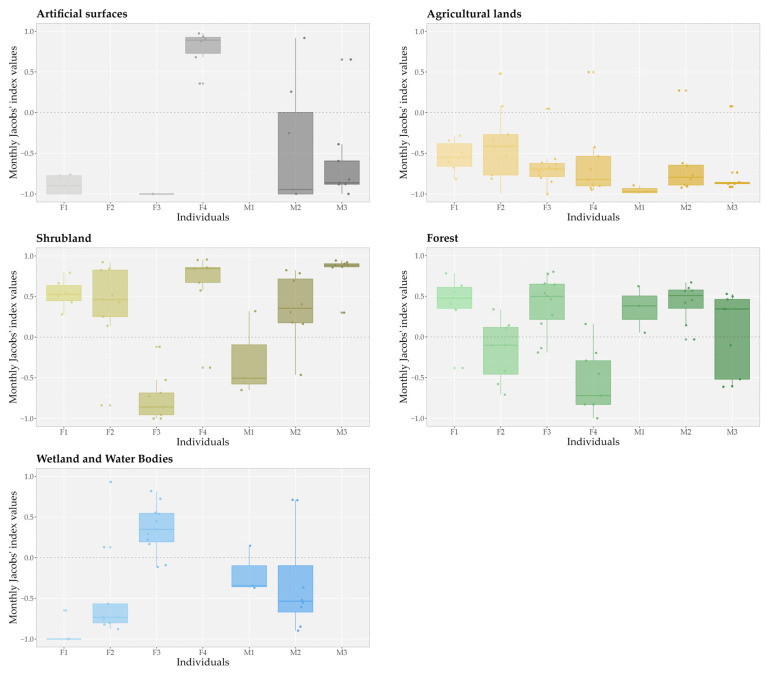
Variety of the habitat preferences of seven GPS-tracked golden jackals (*Canis aureus*) by individuals for artificial surface; agricultural land; shrubland; forest; and wetland and water body habitat types based on monthly Jacobs’ index values of habitat selection. The boxplots represent the distribution of Jacobs’ index values for each individual, where the central horizontal line indicates the median, the box shows the interquartile range (IQR; 25th to 75th percentiles), and the whiskers extend to 1.5 times the IQR. Jittered points depict individual data values, while the dashed horizontal line at zero represents no preference.

However, there were substantial differences between the individuals’ monthly habitat availability, as well as their habitat use across all habitat types except for agricultural lands (Figure 4). Agricultural lands were clearly avoided by all individuals during the study period. On average, artificial surfaces were also avoided by the golden jackals, except for individual F4, which exhibited preference for these areas (*D_F4 art_* = 0.79 ± 0.23). The preference for shrublands and forests displayed greater variability among individuals, while wetlands and water bodies were generally avoided by the individuals in territories where they were present.

**Figure 5:**
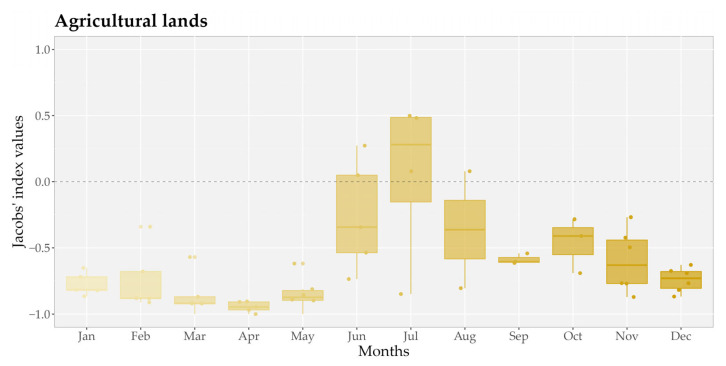
Preference towards agricultural lands by month based on monthly Jacobs’ index values of habitat selection of seven GPS-collared golden jackals (*Canis aureus*). The boxplots represent the distribution of Jacobs’ index values for agricultural lands across months, where the central horizontal line indicates the median, the box shows the interquartile range (IQR; 25th to 75th percentiles), and the whiskers extend to 1.5 times the IQR. Jittered points depict individual data values, while the dashed horizontal line at zero represents no preference.

**Figure A1:**
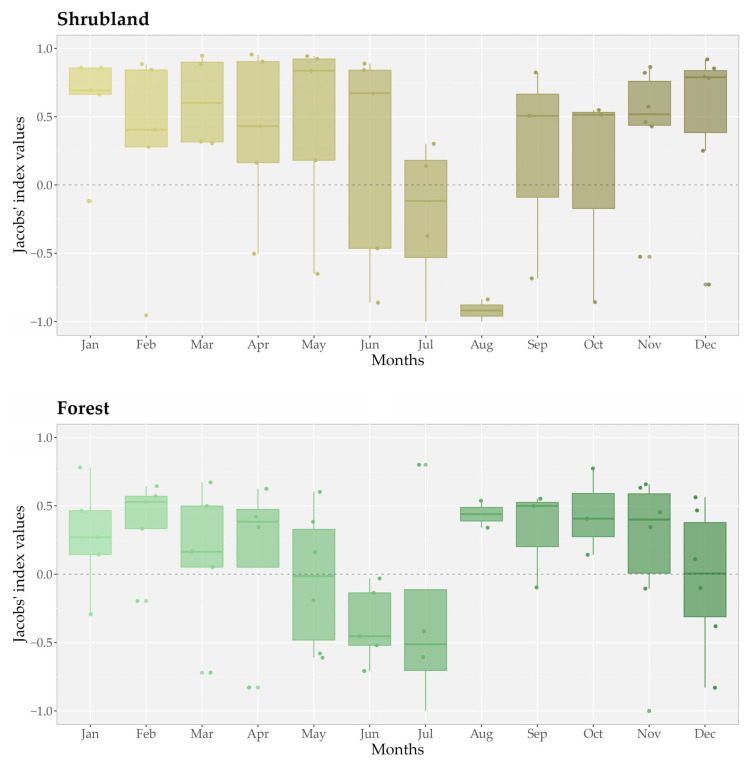
Preference towards shrublands and forests by month based on monthly Jacobs’ index values of habitat selection of seven GPS-tracked golden jackals (*Canis aureus*).

Furthermore, we found that the use of agricultural lands varied among months in general, being highest in the summer, especially in July (*D_agr_* = 0.05 ± 0.63) (Figure 5). However, significant differences were found between the months of spring and summer, as well as between spring and autumn, while an opposite tendency can be detected in the preference towards shrublands and forests (Figure A1).

### 3.3. Use of Ecotones

**Figure 6:**
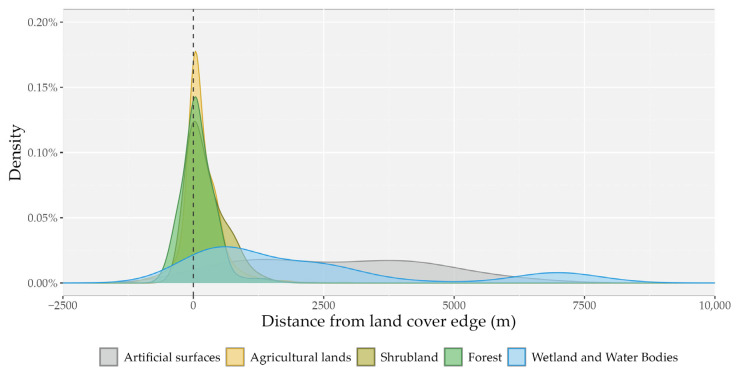
Distribution of distances from landscape feature edges of the localization points (*n* = 29,840) of seven GPS-tracked golden jackals (*Canis aureus*).

We investigated the role of ecotones in the habitat use of the seven tracked golden jackals and found that localization points were primarily recorded at the intersection of forest, agricultural, and shrubland areas. Out of all data points (*n* = 29,840), ~59% were recorded within 250 m of forest and shrubland and ~61% within agricultural land edges (Figure 6). On average, individuals tended to stay outside these habitat patches but most closely to forest ($\bar{x}$ 103 m; range = −704 to 2399 m) and agricultural lands ($\bar{x}$ = 164 m; range = −1506 to 3466 m). Individuals also tended to stay close to the edges of shrublands ($\bar{x}$ = 251 m; range = −572 to 2436 m) but substantially further away from artificial surfaces ($\bar{x}$ = 2748 m; range = −82 to 8049 m) and wetlands and water bodies ($\bar{x}$ = 2336 m; range = −204 to 8224 m). The habitat is fragmented, with a relatively large number of areas close to the edges of forest and agricultural land. However, in the case of forest and shrubland, golden jackals preferentially used interior edges to deeper interiors, and in the case of agricultural lands, they preferentially used exterior edges over greater distances away (Table 4).

**Figure A2:**
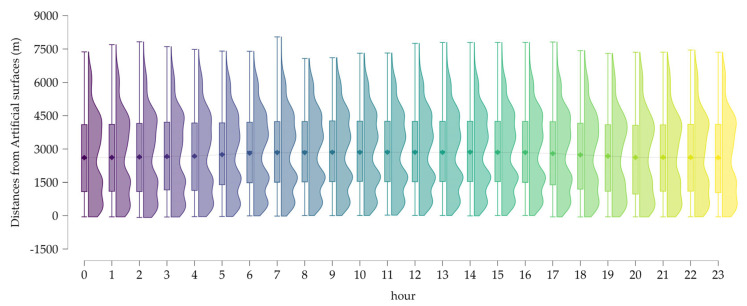

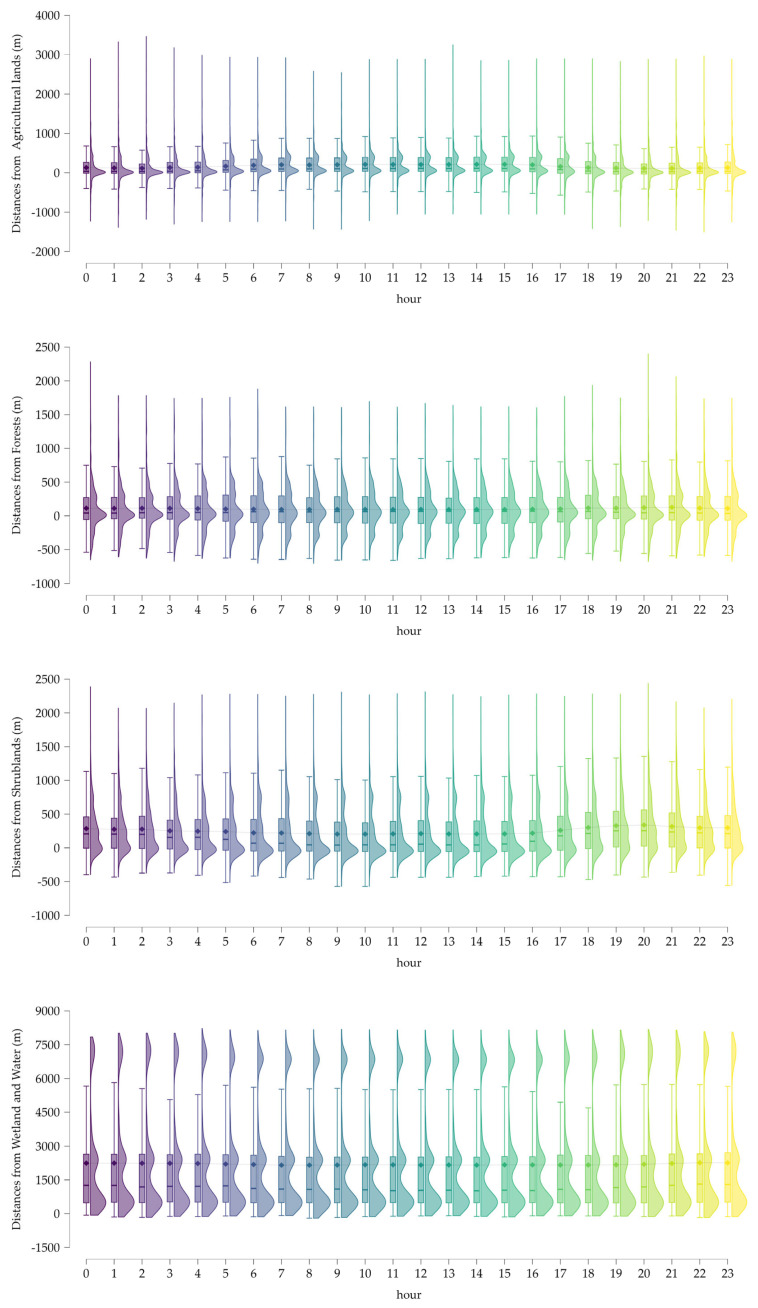
Hourly distances (m) from different landscape features of seven GPS-tracked golden jackals (*Canis aureus*) in the southwestern part of the Pannonian Basin.

We also examined if any circadian patterns characterized the habitat use of the tracked golden jackals [8] but did not find any significant differences between day-time and nocturnal habitat choices (Figure A2).

## 4. Discussion

In our study, we examined the home range sizes and explored the habitat use of golden jackals living in a predominantly agricultural area in Central Europe to better understand how the species adapts its behavior in heavily disturbed environments. We found that the tracked golden jackals maintained smaller home ranges in agricultural landscapes than they did across more pristine environments. They generally preferred vegetation covers provided by forests and shrublands, while they avoided agricultural, arable lands, especially during the breeding and pup-rearing periods. We also found that forest–agricultural ecotones played a significant role in the habitat selection of the tracked golden jackals, as forest edges proved to be the most frequently visited habitats.

### 4.1. Home Range Sizes in Different Environments

Supporting our first hypothesis (H1), the home range sizes of the resident golden jackals tracked across the agricultural landscapes of the southwestern part of the Pannonian Basin (Table A1) were found to be smaller compared to home ranges in more pristine environments (Table 5). Agricultural areas provide abundant food resources that may enable individuals to forage over smaller areas [19,25]. In addition, forest and shrubland habitats across agricultural landscapes are fragmented, resulting in more extensive habitat edges, which provide access to shelter and food resources for golden jackals [7,10,40]. Therefore, the increased availability of suitable habitats in the form of ecotones may also lead to smaller home ranges in agricultural lands.

While previous studies conducted in predominantly agricultural areas also support this argument, some differences among the results may arise from the varying characteristics of these agricultural areas. As shown previously [7], agricultural intensity and land-use patterns influence the habitat selection of golden jackals, which may also manifest in differing home range sizes accordingly.

### 4.2. Habitat Selection Patterns of the Golden Jackal

The results of this study revealed, as predicted, that the tracked golden jackals preferred forests and shrublands (P1) in their habitat selection but with high inter-individual variability, which aligns with the species’ generalist nature. Meanwhile, all individuals strongly avoided agricultural lands supporting our second prediction (P2), especially between February and June, in the breeding and pup-rearing periods. This avoidance might be explained by the overall clear preference for the cover and safety provided by shrublands and forests in the same periods. In addition, while winter crops may provide cover for the animals until being harvested in June and early July, these areas then supply an abundance of food resources in the form of increased densities and/or availability of small mammals, the primary prey of the golden jackal [29,31]. Overall, our findings support the hypothesis that the golden jackal prefers forests and shrublands providing permanent shelter over agricultural lands.

At the same time, golden jackals not only avoided artificial surfaces and wetlands and water bodies but also stayed relatively far from these areas. An exception was the F4 individual, which showed a preference towards areas close to human settlements. On average, two-thirds of its monthly home ranges were covered by agricultural land and less than 1% by artificial surfaces. Hence, even though locations recorded close to human settlements never exceeded 5%, calculations resulted in a preference towards these areas. It is important to add though that 80% of its locations on artificial surfaces were recorded in November and December, when natural food resources become scarcer.

### 4.3. Role of Ecotones in the Habitat Selection of Golden Jackals

According to our results, golden jackals dominantly used the edges of forests, agricultural lands, and shrublands, supporting our fourth prediction (P4). Forest–agricultural ecotones provide proximity to both shelter and food resources [7,10,40]. Based on our results, vegetation cover provided by forests and shrublands is most preferred by the golden jackal during breeding and pup-rearing periods; however, they barely moved away from these habitats throughout the whole study. At the same time, even if golden jackals avoided agricultural lands on average, they still stayed very close to them. A possible reason is that the high diversity and abundance of small mammals in forest–agricultural ecotones increases mammalian predator activity in habitat edges [40]. In addition, by remaining at the edges of agricultural lands, golden jackals may also benefit from the increased availability of small rodents in these areas once winter crops are harvested. Overall, our results support the hypothesis that forest–agricultural ecotones play a key role in the habitat use of golden jackals by providing shelter and improved foraging conditions (H3).

## 5. Conclusions

The habitat use strategy of golden jackals, which involves a preference towards remaining at the edges of forests and agricultural lands, confirms the significance of ecotones, as these areas provide both shelter and proximity to food resources. The use of ecotones and the tendency of the golden jackal to avoid inhabited areas support their expansion and population growth. By relying on ecotones for both food and shelter while steering clear of human-dominated environments, the golden jackal is able to exploit a variety of habitats and minimize human conflict. The use of ecotones strengthens the hypothesis that the diet of the golden jackal follows that of meso-carnivore species and that the presence of small mammals/rodents and small game species of birds and mammals is important for the species. Indeed, ecotones are primarily important for these species in agricultural environments. It also helps the golden jackal to consume regular fruit and fruit crops, including grapes on the edge of vineyards. This adaptive strategy not only facilitates their survival but also promotes population stability and enables their expansion into new areas.

## Figures and Tables

**Table 1:** Summary table of the seven GPS-collared golden jackals (*Canis aureus*) tracked across the southwestern part of the Pannonian Basin.

ID	Sex	Age	No. of Fixes	Deployment Period	Days Tracked
F1	female	juvenile	3238	19 September 2021–1 February 2022	135
F2	female	adult	4846	24 April 2022–30 December 2022	250
F3	female	juvenile	5852	30 May 2022–23 March 2023	297
F4	female	adult	5242	20 November 2022–10 July 2023	232
M1	male	adult	1518	15 March 2021–17 May 2021	63
M2	male	juvenile	4557	6 November 2021–30 June 2022	236
M3	male	adult	4587	25 November 2022–9 July 2023	226

**Table 2:** Home range and core area sizes (in km^2^) of seven GPS-tracked golden jackals in the southwestern part of the Pannonian Basin estimated by the alpha concave hull, 95% minimum convex polygon, and 95% and 50% kernel density estimation methods.

ID	Sex	Age	ACH	MCP95	KDE95	KDE50
F1	female	juvenile	230.6	344.1	373.6	76.6
F2	female	adult	15.9	2.3	2.5	0.3
F3	female	juvenile	18.9	16.6	4.8	0.7
F4	female	adult	21.2	5.0	3.3	0.4
M1	male	adult	13.0	17.1	6.7	0.6
M2	male	juvenile	627.5	624.6	526.8	65.5
M3	male	adult	19.1	5.7	4.8	0.5

**Table 3:** Home range and core area sizes (in km^2^) of a female and male juvenile golden jackal before and after shifting their home ranges to new territories estimated by the alpha concave hull, 95% minimum convex polygon, and 95% and 50% kernel density estimation methods.

ID	No. of Fixes	ACH	MCP95	KDE95	KDE50
Before the home range shift					
F1	1479	128.1	93.5	122.7	25.8
M2	917	216.9	237.6	281.7	61.8
After the home range shift					
F1	1687	63.5	46.3	41.9	5.2
M2	3613	165.4	143.4	119.0	18.0

**Table 4:** Availability and relative use by golden jackals of interior and exterior land or water within 250 m of the edge of landcover types. ‘Use’ is the percentage of radio-telemetry localization points.

Land Cover Categories	Habitat Interiors Within 250 m		Habitat Exteriors Within 250 m	
	Available Habitat	Use of Habitat	Available Habitat	Use of Habitat
Artificial surfaces	6%	0%	12%	3%
Agricultural lands	31%	17%	18%	44%
Shrubland	13%	35%	25%	24%
Forest	7%	24%	17%	35%
Wetland and water bodies	3%	3%	5%	8%

**Table 5:** Published results on the home range size of the golden jackal (*Canis aureus*) across different habitat types.

Habitat Type	Sample Size	Home Range and Core Area Sizes	Calculation Method
Pristine or semi-natural habitats			
Low rolling hills with planted forests dominated by oak, wild Palestine cashew, and Mediterranean Maqui, Israel [25]	8	Home range: 21.2 ± 9.3 km^2^ Core area: 3.5 ± 1.6 km^2^	KDE90 KDE60
Dipterocarp forest, mixed deciduous forest, and dry evergreen forest, Thailand [18]	1	Home range: 26.3 km^2^	MCP95
High forest cover (primarily of English oak) interspersed with agricultural fields and small settlements, Hungary [8]	1	Home range: 66.6 km^2^	MCP95
Dry dipterocarp forest with small patches of mixed deciduous evergreen and riparian forests, Cambodia [34]	6	Home range: 39.6 ± 2.7 km^2^ Core area: was 9.1 ± 1.1 km^2^	MCP95 KDE50
Forest–agricultural habitats and with a high forest cover, Hungary [10]	45	Home range: 14.4 ± 2.3 km^2^	KDE95
Predominantly agricultural landscapes			
Croplands, grasslands, and saline marshes, India [33]	6	Home range: 14.30 ± 4.06 km^2^	MCP95
Agricultural villages (fruit orchards, vineyards, poultry farms), Israel [25]	8	Home range: 6.6 ± 4.5 km^2^ Core area: 1.2 ± 0.9 km^2^	KDE90 KDE50
Agricultural and open fields with forests fragments, hedgerows, and lush vegetation bordering water channels, Serbia [8]	4	Home range: 9.16 ± 7.32 km^2^	MCP95
Agricultural areas of the Po Plain, Italy [19]	8	Home range: 31.22 km^2^ (min: 2.22, max: 135.92) Core area: 3.24 km^2^ (min: 0.15, max: 14.84)	KDE95 KDE50

## Data Availability

The datasets generated and analyzed during the current study are not publicly available but are available from the corresponding author upon reasonable request.

## Appendix A

**Table A1:** Home range and core area sizes (in km^2^) of seven GPS-tracked golden jackals in the southwestern part of the Pannonian Basin estimated by different methods, allowing for comparison with the published literature.

ID	Sex	Age	MCP100	MCP95	MCP50	ACH	KDE95	KDE90	KDE60	KDE50
F1	female	juvenile	382.5	344.1	194.5	230.6	373.6	283.8	105.8	76.6
F2	female	adult	21.0	2.3	0.3	15.9	2.5	1.7	0.4	0.3
F3	female	juvenile	23.3	16.6	0.6	18.9	4.8	3.1	0.9	0.7
F4	female	adult	27.8	5.0	0.6	21.2	3.3	2.3	0.6	0.4
M1	male	adult	18.7	17.1	0.3	13.0	6.7	3.9	0.9	0.6
M2	male	juvenile	719.9	624.6	115.7	627.5	526.8	384.9	99.7	65.5
M3	male	adult	25.1	5.7	0.5	19.1	4.8	3.5	0.7	0.5
